# Certified Coffee: Does the Premium Pay Off?

**DOI:** 10.1289/ehp.115-a456

**Published:** 2007-09

**Authors:** David A. Taylor

Coffee bean prices plummeted during the coffee crisis of the late 1990s, the result of a glut in coffee production. Prices sank from around US$1.50 per pound in 1997 to about a third that amount in 2001, according to the International Coffee Organization, the primary intergovernmental organization for coffee production. For millions of people dependent on coffee farming, the crisis brought social and economic devastation and forced many farmers to choose between immediate household needs and environmental destruction.

That crisis raised alarms in Europe that contributed to the rise of Fair Trade certification for coffee, intended in part to give coffee farmers a buffer against market fluctuations. Fair Trade and other programs designed criteria for certifying the production of items ranging from organic foods and coffee to timber and pulp, with one aim being to reward better management of forests with a premium for proof of sustainable management. When farmers have a buffer against market fluctuations, they will be less likely to choose forest destruction when prices go down. Now researchers and journalists are asking whether certification is making a difference in the health of people and the forests where these products originate.

## Seeing the Forest for the Beans

Coffee beans, the world’s second largest traded commodity, is a major cash crop for millions, especially in Latin America, where most coffee bushes grow shaded by trees. In some places, coffee is grown under managed tree cover, meaning the shade trees are specially planted and trimmed. The shade cover can be sparse in some areas. For example, in El Salvador, shade cover varies between 40% in the hotter lower altitudes (where more shade is needed to retain soil moisture) to 20% at higher altitudes. Some coffee (“sun coffee”) is grown without any tree cover at all.

In Oaxaca and other parts of Mexico, the natural forest canopy shades the coffee bushes. In 2001, during the period that Allen Blackman calls “the trough of the coffee crisis,” he stood in a town in Oaxaca, looking up at the forested hillsides where shade-grown coffee grew. What he saw was alarming.

“You could look up into the hills around Puerto Angel and see big patches of cleared forest,” recalls Blackman, a senior fellow at Resources for the Future (RFF), a nonprofit think tank devoted to environmental research. Townspeople in southern Oaxaca were talking about the forest cutting and the consequences of erosion and siltation of water channels, but research was scarce.

Blackman proposed studying what was going on and received funding from the Tinker Foundation and the Commission for Environmental Cooperation. What he and his RFF colleagues would find was that farmers were clearing trees to plant corn and beans so their families could survive the coffee market crash. In time, he would further learn that, between 1993 and 2001, clearing had destroyed 3% of the area’s forest—about 8,000 hectares, roughly half the size of Washington, DC.

## The Beginning of Certification

From a start in Europe in 1988, an international umbrella for Fair Trade certification was established in 1997. Fair Trade stipulates that certified cooperatives receive a premium of at least US$0.10 more per pound for their products than uncertified farms; that amount can be greater, depending on market conditions.

Initial indications suggest that certification has had a beneficial effect on forest health. A 22 April 2007 article in *The New York Times* by Elisabeth Malkin described Fair Trade and other certification programs as having given Mexican coffee growers an incentive to save trees that protect hillsides against erosion and maintain watershed quality. Farmers on the slopes of the Sierra Madres told Malkin the higher prices paid for certified coffee beans helped maintain their cooperatives and their interest in growing coffee.

“Fair Trade comes into its own when the coffee market is in crisis,” says Luke Upchurch, head of media for Consumers International. This consumer advocacy organization based in London that commissioned *From Bean to Cup*, a 2005 study of the coffee industry.

The idea of certification has flourished among consumers. Among the four out of five Americans who call themselves coffee drinkers, awareness of Fair Trade certification more than doubled from 12% in 2004 to 27% in 2007, according to the National Coffee Association, an industry group. Awareness of Organic certification rose from 45% in 2004 to 54% two years later.

In 2005, sales of all Fair Trade products—not just coffee—reached about US$850 million, according to figures from Fairtrade Labelling Organizations International, an umbrella entity that unites labeling initiatives and producer networks in Central and South America, Africa, and Asia. About 400 companies purchase at least a portion of their coffee under Fair Trade terms. For products with Organic certification, which has been growing for two decades, total retail sales are much higher: US$20 billion a year. Starbucks and several other companies have their own internal programs for certification.

Rodney North, information officer with Equal Exchange, a leading U.S. importer, says that company, like some other importers, is committed to buying both Organic and Fair Trade. North describes how the two systems relate: Fair Trade emphasizes social indicators, whereas the Organic label stresses environmental criteria. Still, the two programs overlap. For example, besides fair wages and safe working conditions, Fair Trade requires farmers to minimize their use of agricultural chemicals, dispose of waste safely, and maintain soil and water quality. Fair Trade farmers can’t use chemicals branded as the Dirty Dozen due to hazards they pose to human or environmental health; these include the pesticides chlordane, heptachlor, DDT, aldrin, endrin, lindane, parathion, and methyl parathion. Fair Trade also bars a handful of other pesticides highlighted by the UN Food and Agriculture Organization in all steps of production, from planting through postharvest processing.

*From Bean to Cup* notes that coffee certification has prompted better health and safety measures for coffee workers by requiring that they use protective clothing and equipment when applying chemicals. Many farmers told study investigators that the reduced use of pesticides had benefited local health and well-being, according to Upchurch. However, the report also notes that workers sometimes resist the use of protection, complaining of discomfort.

By providing a price premium, certification also gives coffee growers an economic basis for investing in community health. *From Bean to Cup* estimates that during the worst of the coffee crisis, the economic losses in Latin America amounted to US$4.5 billion per year, plus losses to children’s education and health care as economic losses meant fewer school resources and family cutbacks on health care expenses. Some of the cooperatives involved with Fair Trade invested the premium paid for certified coffee directly in public improvements. Santiago Arguello, who manages coffee certification programs in Mexico for Agroindustrias Unidas, a subsidiary of the multinational ECOM Agroindustrial Corporation, gives two examples: one village in Mexico invested in the local school, and a cooperative in Guatemala built a local clinic.

Arguello, himself a third-generation coffee grower, buys coffee from farmers in southern Mexico, including Oaxaca and Chiapas. Although overall demand for coffee from those areas is declining, he says, demand for certified coffee there continues to rise. In his view, the main benefits of certification are market stability and quality standards. “We believe that we’re helping farmers define the long-term vision for their businesses,” he says.

## Qualifying the Bean Buzz

Some researchers say the jury is still out on whether certification helps the environment. Stacy Philpott, an environmental scientist at the University of Toledo, has studied the effects of coffee certification on biodiversity as an indicator of environmental health. In 2004 and 2005, with funding by the Smithsonian Migratory Bird Center, Philpott and colleagues investigated biodiversity as measured in the number of species on farms in coffee-growing cooperatives in Chiapas. They divided cooperatives into three categories: farms certified as Organic, farms with both Organic and Fair Trade certification, and farms that had no certification.

Their report, published in the August 2007 issue of *Conservation Biology*, showed socioeconomic differences among the farm types that could affect health. Organic-certified farms, for example, tended to grow more varieties of produce, especially fruits, for their own consumption—a fact that could hold an important linkage to human health and nutrition. Fair Trade farms fared a bit better economically during the worst times, and tended to invest more in local schools and food processing facilities. But Philpott found no difference in biodiversity.

“The take-home message was, there was no difference in terms of biodiversity,” she says. She was also skeptical of the claim that certification prevents farmers from shifting to another crop when coffee prices plummet. “I was really surprised at how few farmers had changed anything [in response to coffee price changes],” she says. “They’re so culturally attached to coffee.” Still, she notes, the study sample size was very small—just 10 cooperatives—and certification programs may yet help to maintain biodiversity under the canopy that shade coffee requires.

In Oaxaca, however, Blackman and his RFF colleagues found that land use in coffee-growing areas did change in response to falling coffee prices. Farmers were clearing trees in order to plant subsistence crops because their income from coffee was not sufficient to support their families. The cleared patches did not necessarily replace coffee; rather, many farmers were clearing in forested areas near their farms where coffee was not growing.

Through a combination of informal focus groups and satellite image analysis, Blackman and his colleagues assembled a picture suggesting that rock-bottom coffee prices corresponded with a shift from forest cover to subsistence crops during the 1990s. Patches of cleared forest like those he saw around Puerto Angel were often planted with corn and beans for several years before the soil was depleted. “We don’t have a farm-level survey,” Blackman admits, “but that appears to be what’s going on.”

His research in Mexico found that policies that promoted farmer marketing cooperatives, sometimes thought to undermine natural forest conservation, can actually sometimes help preserve tree cover for shade coffee and other nontimber crops. Inasmuch as Fair Trade and other certification programs promote well-run cooperatives, they can promote land use stability.

Blackman and colleagues also looked at El Salvador, which lost 13% of its tree cover in shade coffee areas between 1990 and 2000. They hypothesized that certification and direct payments were not likely to be effective in stemming tree clearing in the western and center regions of the country, where most Salvadoran coffee is grown. Land prices in these areas were so high that farmers reaped big profits from selling farms to developers, and neither certification nor direct payments were likely to be large enough to make a difference in this calculation. “That said,” says Blackman, “certification and direct payments could make a difference in some parts of El Salvador, like the east where a lot of clearing is due to subsistence agriculture and not so much to urbanization.” What was needed, he concluded, was for the government to more vigorously enforce restrictions on land use changes.

The best prospect for coffee growers, in Blackman’s view, may rest less on certification and more on producing top-quality coffee. Agro-climatic factors for premium coffee—shade forests at high elevations—favor Latin American countries. With better farm practices, the region’s growers are capitalizing on that natural advantage. Certification may help, says Blackman, but in the marketplace “the really significant premiums come from quality, whether you’re certified or not.”

## Certifiably Sound

Certification programs for other forest products may also hold lessons for the coffee industry and policy makers. Gary Dunning, a program director at Yale’s School of Forestry and Environmental Studies, has for several years managed an international dialogue on certification systems for wood involving key private, public, and nonprofit representatives. There are a number of national and international forest certification systems out there now. One of the most respected, says Dunning, is managed by the Forest Stewardship Council (FSC), an international nonprofit organization that encourages responsible forest management. Besides the FSC’s program for certifying wood and paper, it is exploring certification of bamboo and other nonwood items, including Brazil nuts.

“Certification isn’t just about certifying the tree and its products,” says Karen Steer, a member of the FSC board. “It’s about certifying the whole forest.” In that sense, it can be a tool adapted for different products to promote forest management that is sustainable in its ecologic, economic, and social indicators. Health and social values are embedded in the contract that each producer signs with the FSC, says Steer. The council uses two types of assessments: a forest management assessment and a chain-of-custody document. The first assesses the environment before the product leaves the forest; the second documents conditions encountered at each stage after the item leaves the forest.

Steer witnessed certification at work in Bolivia, where she spent four months in late 2005. There she saw that Fair Trade–certified harvesters of Brazil nuts enjoyed a more stable income and steadier demand—and were therefore less likely to overharvest from the forest—than their noncertified counterparts. She also saw that human well-being and forest health are clearly intertwined, as evidenced by village clinics being established with proceeds from a healthier Brazil nut habitat and stable supply, and families being able to afford treatment.

Joshua Rosenthal, deputy director of the international research division at the NIH Fogarty International Center, views that linkage through the lens of infectious disease and its transmission. In general, diverse old-growth forests tend to buffer the effects of various infectious diseases. “Deforested areas that have allowed numerous invasive species to establish themselves tend to have higher infection rates,” says Rosenthal; they are more likely to harbor animals that can be reservoirs or vectors for spreading disease and reduce the landscape’s ability to retain and purify water. “To the extent that certification does contribute to maintaining healthy, diverse forests, you’re likely to have reduced risk of infectious disease.”

Steer sees a trend toward harmonizing various certification programs that can sometimes have confusing overlap and differences. Arguello says the farmers he works with would welcome that clarification. Many of the practices required for the different labels are the same, these farmers say, so why haven’t certifiers created a common code?

## Buyer Be Aware

Consumer education doesn’t directly affect coffee-growing communities, but its value shouldn’t be ignored, says North. Consumers may be first drawn to buy certified food products out of concern for their personal health, but they then become curious about conditions up the supply chain. That way, he says, certification has a “ratcheting effect” on buyer awareness.

Dunning affirms that U.S. consumer education can have an impact on global forest conservation. “Europe has gone a long way to monitor its supply stream,” he says, speaking of the timber market. “Being the largest consuming nation, the United States has the biggest role to play, which the American public hasn’t fully realized yet.”

## Figures and Tables

**Figure:**
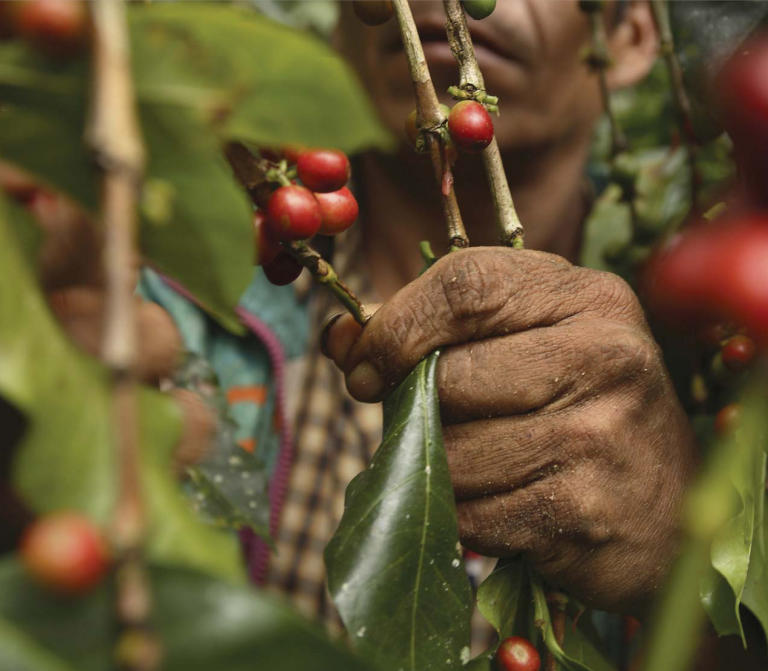
Making the best Pedro Gonzalez, 40, picks ripe coffee on a small coffee farm associated with the Las Brumas Cooperative in Nicaragua. The cooperative is part of the larger Organization of Northern Coffee Cooperatives, which helps members market their coffee and negotiates better prices with wholesalers. Farmers with the Los Brumas Cooperative have used Fair Trade premiums to build a school and improve roads in their town.

